# Shoot- and root-borne cytokinin influences arbuscular mycorrhizal symbiosis

**DOI:** 10.1007/s00572-016-0706-3

**Published:** 2016-05-19

**Authors:** Marco Cosme, Eswarayya Ramireddy, Philipp Franken, Thomas Schmülling, Susanne Wurst

**Affiliations:** 1 Functional Biodiversity, Dahlem Centre of Plant Sciences, Institute of Biology, Freie Universität Berlin, Königin-Luise-Straße 1-3, 14195 Berlin, Germany; 2 Department of Plant Propagation, Leibniz Institute of Vegetable and Ornamental Crops, Kühnhäuser Straße 101, 99090 Erfurt-Kühnhausen, Germany; 3 Plant-Microbe Interactions, Department of Biology, Faculty of Science, Utrecht University, PO Box 800.56, 3508 TB Utrecht, The Netherlands; 4 Applied Genetics, Dahlem Center of Plant Sciences, Freie Universität Berlin, Albrecht-Thaer-Weg 6, 14195 Berlin, Germany

**Keywords:** *Nicotiana tabacum*, *Rhizophagus irregularis*, Symbiosis, Cytokinin, Carbon, Phosphate transporter genes

## Abstract

The arbuscular mycorrhizal (AM) symbiosis is functionally important for the nutrition and growth of most terrestrial plants. Nearly all phytohormones are employed by plants to regulate the symbiosis with AM fungi, but the regulatory role of cytokinin (CK) is not well understood. Here, we used transgenic tobacco (*Nicotiana tabacum*) with a root-specific or constitutive expression of CK-degrading *CKX* genes and the corresponding wild-type to investigate whether a lowered content of CK in roots or in both roots and shoots influences the interaction with the AM fungus *Rhizophagus irregularis*. Our data indicates that shoot CK has a positive impact on AM fungal development in roots and on the root transcript level of an AM-responsive *phosphate transporter* gene (*NtPT4*). A reduced CK content in roots caused shoot and root growth depression following AM colonization, while neither the uptake of phosphorus or nitrogen nor the root transcript levels of *NtPT4* were significantly affected. This suggests that root CK may restrict the C availability from the roots to the fungus thus averting parasitism by AM fungi. Taken together, our study indicates that shoot- and root-borne CK have distinct roles in AM symbiosis. We propose a model illustrating how plants may employ CK to regulate nutrient exchange with the ubiquitous AM fungi.

**Electronic supplementary material:**

The online version of this article (doi:10.1007/s00572-016-0706-3) contains supplementary material, which is available to authorized users.

## Introduction

The arbuscular mycorrhizal (AM) symbiosis, a widespread association between terrestrial plants and fungi of the phylum Glomeromycota, is regulated from the plant side by nearly all known phytohormones (Bucher et al. 2014; Gutjahr 2014; Pozo et al. 2015). Cytokinins (CK) constitute a class of phytohormones that regulates many fundamental aspects of plant development (Kieber and Schaller 2014; Werner and Schmülling 2009). Plants colonized by AM fungi generally have enhanced CK levels in both shoots and roots compared with non-AM (NAM) plants (Allen et al. 1980; Barker and Tagu 2000; Shaul-Keinan et al. 2002). Yet, the function of CK in AM symbioses is poorly understood.

The AM fungi are obligate biotrophs that obtain photosynthetically fixed carbon (C) in colonized roots via transport of sugars from root cortex cells to the intracellular arbuscules in the apoplast of the root cortex cells and to the intercellular hyphae (Helber et al. 2011; Smith and Read 2008). In exchange, the AM mycelium spreading into the soil takes up mineral nutrients particularly phosphorus (P), along with nitrogen (N) and other minerals, transports them through their hyphae towards the root, and delivers them via the arbuscules into the surrounding plant cells (Marschner 1995; Nouri et al. 2014). The mycorrhizal delivery of P is regulated by the root through particular *phosphate* (Pi) *transporter* (*PT*) genes (Maeda et al. 2006; Nagy et al. 2005). The direct pathway for Pi uptake via root hairs and epidermis may be, in some cases, suppressed by AM colonization (Chen et al. 2007; Grunwald et al. 2009; Javot et al. 2007). Despite the reciprocal nutritional benefit, the plant growth response to AM colonization can range from positive to negative according to a mutualism-parasitism continuum (Johnson 2010). Mutualistic benefits of delivered P are predicted to be the greatest in soils that are characterized by high N and low P availability (Johnson 2010). By contrast, when neither N nor P is limited, fungal growth is primarily limited by C, so the fungal C demand can increase to the point where it may depress plant growth and generate fungal parasitism (Johnson 2010). Other factors such as fungal genotype, competition, or complementarity can influence as well the AM symbiotic outcome (Angelard et al. 2010; Engelmoer et al. 2014; Jansa et al. 2008; Maherali and Klironomos 2007).

The study of mutants and transgenic plants altered in phytohormone metabolism or perception has provided major insights into their role in the regulation of AM symbiosis. The arbuscule formation vital to the net benefits of AM symbiosis is regulated positively by abscisic acid (ABA), via a dual ethylene-dependent⁄ethylene-independent mechanism (Martín-Rodríguez et al. 2011). In contrast to ABA, gibberellic acid (GA) negatively regulates arbuscule formation via degradation of DELLA proteins, which are required for arbuscule formation (Floss et al. 2013; Foo et al. 2013; Yu et al. 2014). Jasmonate positively regulates arbuscule formation but negatively regulates fungal spread inside the roots, possibly by restricting the C allocated to colonized roots or by increasing root defenses (Gutjahr et al. 2015; Wasternack and Hause 2014). Salicylic acid slows down fungal growth without changing final colonization (Herrera-Medina et al. 2003). Absence of brassinosteroids do not appear to affect directly the arbuscule formation but decreases AM hyphal colonization (Bitterlich et al. 2014). Auxin appears to enhance the hyphal colonization, and auxin perception is required for arbuscule formation (Etemadi et al. 2014; Hanlon and Coenen 2011). In pea (*Pisum sativum*), the auxin effect was partially mediated by strigolactones (Foo 2013), which in turn stimulate the presymbiotic hyphal branching and enhance the number of infection points in the roots (Yoshida et al. 2012). Altogether, it is evident that plants can employ an extensive phytohormone network to regulate their symbiotic interaction with AM fungi.

Much less is known about the regulatory role of CK in AM symbioses. CK are N^6^-substituted purine derivatives that regulate many fundamental aspects of plant development, including photosynthesis and root uptake of mineral nutrients (Kieber and Schaller 2014; Werner and Schmülling 2009). The positive effects of AM fungi on the CK levels of the host plant (Allen et al. 1980; Baas and Kuiper 1989; Barker and Tagu 2000) appears to be uncharacteristic of pathogenic fungi (van Rhijn et al. 1997) and independent from the increased P supply in roots (Shaul-Keinan et al. 2002; Torelli et al. 2000). Drüge and Schonbeck (1992) observed a strong correlation between increased CK levels, improved photosynthesis, and enhanced growth of AM plants and hypothesized that CK is part of the positive AM effect on plant performance. However, this hypothesis has been contested as the correlation was not always observed (Baas and Kuiper 1989; Danneberg et al. 1993). Similarly, contradictory results have been found in genetic studies. For example, the *cre1* mutation in *Medicago truncatula*, which causes reduced CK perception, had no effects on AM colonization (Laffont et al. 2015). In contrast, an increased AM colonization was observed in CK-deficient *35S*:*CKX2* transgenic tobacco (*Nicotiana tabacum*) (Cosme and Wurst 2013) as well as in the CK-overproducing E151 pea mutant (Jones et al. 2015). Thus, CK appears to have an elusive role in AM symbiosis, and it is yet unclear why AM plants generally have enhanced CK levels in both shoots and roots (Barker and Tagu 2000).

Because CK has opposite roles in shoot and root development (Werner et al. 2001, 2003), an adequate genetic approach to study the role of CK in AM symbiosis is to use plants with distinct contents of CK in these organs. A useful tool to generate plants with a lower CK content has been the ectopic expression of cytokinin oxidase/dehydrogenase (*CKX*) genes (Werner et al. 2001, 2003, 2010). *CKX* genes code for enzymes that irreversibly degrade CK in a single enzymatic step to biologically inactive molecules (Schmülling et al. 2003). Constitutive expression of *CKX* genes under the control of the *35S* promoter reduced the levels of CK in the whole plant, which positively affects root growth, but strongly reduces shoot growth as well as leaf chlorophyll and sugar content (Werner et al. 2001, 2003, 2010). In order to generate plants with a reduced CK content particularly in roots, enhanced *CKX* expression has been limited mainly to the roots by using the tobacco root-specific promoter *WRKY6* (Werner et al. 2010). Leaves of WRKY6:CKX1 transgenic plants (hereafter designated as W6:CKX1) exhibited levels of bioactive CKs similar to the leaves of wild-type (WT), whereas the levels of bioactive CKs in the roots were reduced by ∼30 % (Macková et al. 2013). Consequently, shoot development of W6:CKX1 plants was very similar to that of the WT, while the size of the root system was increased by 27–39 % (Macková et al. 2013; Werner et al. 2010).

In the present study, we tested whether a reduction of CK levels restricted mainly to the roots influences the plant interaction with AM fungi and whether the shoot CK status affects AM symbiosis as well. To this end, we compared the AM colonization and plant performance of W6:CKX1 transgenic tobacco plants with that of the untransformed WT and two transgenic lines with constitutive reduction of CK levels (35S:CKX1, 35S:CKX2). Furthermore, we tested whether AM fungal strains or their interaction influence the symbiotic outcome by using single and simultaneous inoculation with two different strains of *Rhizophagus irregularis* (formerly *Glomus intraradices*). Potential functional mechanisms underlying plant and fungal responses were explored by determining the P, N, and C content in shoots and the transcript levels of *PT* genes in roots.

## Materials and methods

### Plant and fungus

*Rhizophagus irregularis* (Błaszk., Wubet, Renker and Buscot) Walker and Schüßler (former *Glomus intraradices*) is a widespread AM fungus and the first one that has been used for large-scale transcriptome sequencing (Tisserant et al. 2012). Although it is generally effective in colonizing roots and transferring mineral nutrients to the host plant, *R. irregularis* symbiotic function may vary with strain genotype (Angelard et al. 2010). To unveil eventual strain effects, we used two different *R. irregularis* strains (RI and FM) obtained from INOQ GmbH (Soltau, Germany). Inocula of *R. irregularis* were produced in sand using mixed plant cultures of *Plantago lanceolata*, *Tagetes erecta*, and *Zea mays*, and contained 200 propagules mL^−1^, consisting of spores, hyphae, and colonized root pieces.

As a host plant, we used tobacco (*N. tabacum* L. cv. Samsun NN). The untransformed control is referred to as WT. The transgenic lines expressing *W6*:*CKX1* (line W6-CKX1-24), *35S*:*CKX1* (line 35S:CKX1-50) and *35S*:*CKX2* (line 35S:CKX2-38) were described previously (Macková et al. 2013; Werner et al. 2001, 2008, 2010). Briefly, the W6:CKX1 tobacco line harbors the *CKX1* gene of *Arabidopsis thaliana* (L.) Heynh. under the transcriptional control of the predominantly root-expressed *WRKY6* promoter and has lower levels of bioactive CK mainly in roots (Macková et al. 2013; Werner et al. 2010). The 35S:CKX1 and 35S:CKX2 plant lines harbor two different *CKX* genes (*CKX1* and *CKX2*, respectively) of *A. thaliana* under the transcriptional control of the constitutively expressed *35S* promoter (Werner et al. 2001). Constitutive reduction of the CK content in 35S:CKX1 and 35S:CKX2 lines causes in addition to root enhancement a reduced shoot growth because CK is a positive regulator of shoot growth. Both lines differ in the expressivity of the phenotypic traits, with *35S*:*CKX1* expression causing stronger negative effects on shoot growth than *35S*:*CKX2*, which is reflected by reduced photosynthesis and a lower content of soluble sugar (Werner et al. 2001, 2008).

### Experimental set up

To test whether the root CK status influences the AM symbiosis, and eventually, if the CK status of the shoot has a role as well and whether AM fungal strains and/or their interaction influence the symbiotic outcome, we conducted a factorial experiment in a glasshouse (16-h light and 20/24 °C night/day temperatures). The factors were the tobacco line (four levels: WT, W6:CKX1, 35S:CKX1, 35S:CKX2); *R. irregularis* RI (two levels: −, +); and *R. irregularis* FM (two levels: −, +); which sums up in a full factorial design to 16 treatments, each treatment with 10 independent replicates. The experimental replicate consisted of a plastic pot (2 L) filled with an autoclaved (121 °C, 20 min) soil/sand mixture (1:1, *v*/*v*) described previously (Cosme et al. 2014). Briefly, the soil contained 6.9 mg/100 g P (calcium/acetate/lactate) and 0.12 % of N (C/N analyzer). We inoculated the pots according to AM treatment by mixing thoroughly 100 mL of inoculum on the top layer of the soil/sand mixture. Non-AM (NAM) control pots received sterilized inoculum (autoclaved at 121 °C for 20 min), the *R. irregularis* RI and FM pots received their respective strain inoculum, and the co-inoculated pots received 100 mL of a mixture (1:1, *v*/*v*) of both strain inocula. Additionally, the pots received a microbial wash (20-μm sieve) produced from fungal inocula to correct for the potential presence of NAM microbial backgrounds. All tobacco seeds were surface-sterilized in 1.2 % NaClO for 5 min and rinsed with H_2_O prior use. Each pot was sown with several seeds of the respective tobacco line and immediately after germination the seedlings were thinned, allowing only one single plant to grow in each pot. Plants were watered regularly and fertilized every week with double strength of a modified Hoagland’s solution without P (no. 3 as described by Douds and Schenck 1990) to facilitate AM colonization. After 8 weeks of growth, the number of flowers per plant was counted and all plants were harvested by cutting the shoot at the ground level. The soil was carefully washed away from roots and root sub-samples (0.3 g) were instantly frozen in liquid N_2_ and stored in −80 °C to analyze transcript levels as described later. The shoots and roots were dried in an oven during 1 week at 60 °C and subsequently, their biomasses were recorded.

### AM fungal colonization

To evaluate whether an altered CK content affects AM fungal development, we assessed the percentage of root length colonized by AM hyphae and arbuscules in all experimental plants. To this end, random samples of 10 2-cm-long root fragments were collected from each root system and stained using the ink and vinegar method (Vierheilig et al. 1998). The percent of root length colonization was determined at the microscope (×200 magnification) using the magnified intersections method with 100 intersects per sample (McGonigle et al. 1990).

### Content of phosphorus, nitrogen and carbon in shoots

To determine whether altered plant growth following *R. irregularis* colonization was correlated with an altered nutrient content, we determined the concentration and total content of N and P as well as the concentration of C in shoots. The dried shoots of each plant were homogenized by grinding to fine particles using a ball mill (MM 400, Retsch, Haan, Germany). Sub-samples (ca. 3 mg) of all ground shoots were placed into individual zinc capsules and the percentages of N and C in shoots were determined by standard procedures using a CN Elemental Analyzer (Euro EA, HEKAtech GmbH, Germany), with acetanilide as standard (HEKAtech M.135.17). The concentration of P was determined from ground shoots of five randomly selected plant replicates per treatment, with two technical repetitions (200 mg each) per plant replicate. Technical repetitions were microwave-digested in 5 mL of 65 % HNO_3_ and 3 mL of 30 % H_2_O_2_ using a MARSXpress (CEM GmbH, Kamp-Lintfort, Germany). The digested solution was filtered and diluted with H_2_O in 50-mL volumetric flasks, and P was measured photometrically following the DIN EN ISO 15681–1 norm and using a FIA modula (Medizin- und Labortechnik Engineering GmbH, Dresden, Germany).

### Root transcript levels of phosphate transporter genes

Total RNA was extracted from roots of 8-week-old tobacco plants with the TRIzol method as described by Brenner et al. (2005). RNA was further purified by using RNeasy mini-columns including the on-column DNase digestion as described in the manufacturer’s protocol (appendix D of the QIAGEN RNeasy Mini Handbook, QIAGEN GmbH, Hilden, Germany). Equal amounts of starting material (1 μg of RNA) were used for complementary DNA synthesis using SuperScript III Reverse Transcriptase. Real-time PCR using FAST SYBR Green I technology was performed on an ABI PRISM 7500 sequence detection system (Applied Biosystems Inc., California, USA) and universal “FAST” cycling conditions (10 min at 95 °C, 40 cycles of 15 s at 95 °C and 60 s at 60 °C) followed by the generation of a dissociation curve to check for specificity of the amplification. Gene expression data were normalized against two different reference genes (*N. tabacum* elongation factor 1α (*EF*-*1α*), and *L25* ribosomal protein) according to Vandesompele et al. (2002) and are presented relative to the control treatment. Primers used for reference genes and genes of interest are listed in Supplemental Table 1.

### Statistical analyses

We analyzed AM fungal colonization by two-way ANOVA using only inoculated plants with the categorical factors tobacco line (four levels: WT, W6:CKX1, 35S:CKX1, or 35S:CKX2) and AM inoculation (three levels: *R. irregularis* RI, *R. irregularis* FM, or both RI and FM), since we did not observe colonization in NAM plants and ANOVA is not valid with zero variances. Plant parameters were analyzed by three-way ANOVA with the categorical factors tobacco line (same levels as earlier), *R. irregularis* RI (two levels: −, +) and *R. irregularis* FM (two levels: −, +). Data were tested for normality of errors using Kolmogorov-Smirnov tests and for homogeneity of variances using Levene’s tests. In the case of non-normality and/or unequal variances, data were log or arcsine transformed prior to ANOVA. Multiple comparisons were analyzed by Duncan’s multiple range test. All data were analyzed in R Studio 0.97.332 (www.rstudio.com).

## Results

### AM fungal colonization

**Fig. 1:**
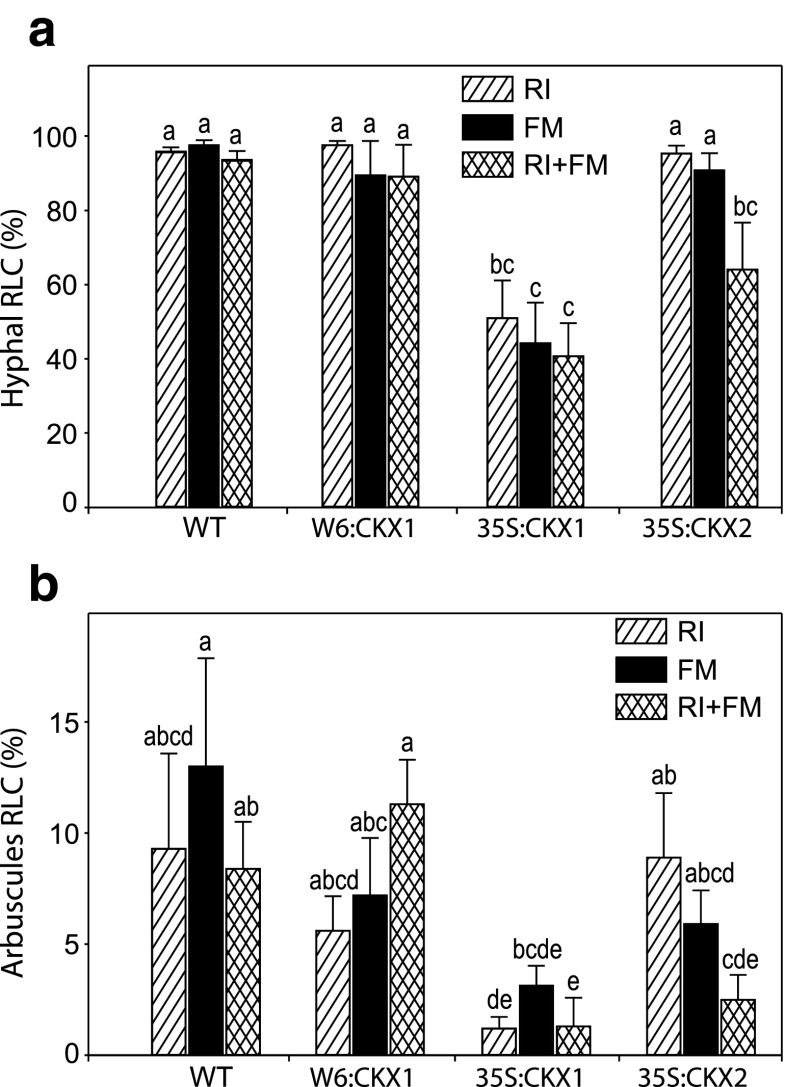
Effects of root-specific and constitutive reduction of cytokinin (CK) levels on arbuscular mycorrhizal (AM) fungal colonization. The transgenic tobacco line W6:CKX1 with a root-specific reduction of CK levels, the 35S:CKX1 and 35S:CKX2 transgenic lines with constitutive reduction of CK levels and the corresponding wild-type (WT) were inoculated with the AM fungus *Rhizophagus irregularis* strain RI, strain FM, or both (RI + FM). Plants were grown in a glasshouse for 8 weeks and sampled to determine the percentage of root length colonization (RLC) by the AM fungal hyphae and arbuscules. Values are means + SE, *n* = 10. For each AM fungal parameter, *bars topped by the same letter* are not significantly different (*p* < 0.05) according to Duncan’s multiple range test

To assess AM fungal development, we quantified the internal hyphal and arbuscules colonization in stained roots of 8-week-old tobacco plants (Fig. 1a, b). No evidence was found for AM fungal colonization in plants treated with sterile inocula (NAM controls). The AM hyphal colonization in WT plants was remarkably high (close to 96 %) and did not differ among fungal inoculations (Fig. 1a; Supplemental Table 2). The lowered CK content in roots (W6:CKX1) did not affect the AM hyphal colonization compared with that of WT (Fig. 1a). In contrast, plant lines with constitutive reduction of the CK content (35S:CKX1 and 35S:CKX2) showed reduced AM hyphal colonization compared to the WT depending on genotype and fungal inoculation. In the case of 35S:CKX1 transgenic plants, a strongly reduced hyphal colonization was observed following all fungal inoculations (from 96 to 45 %), while 35S:CKX2 plants showed reduced hyphal colonization (64 %) only in the fungal co-inoculation treatment (Fig. 1a). Finally, the arbuscule colonization was relatively low across all plant lines and AM fungal inoculations, but followed a similar pattern as the hyphal colonization (Fig. 1b; Supplemental Table 2).

### Plant biomass and fitness

**Fig. 2:**
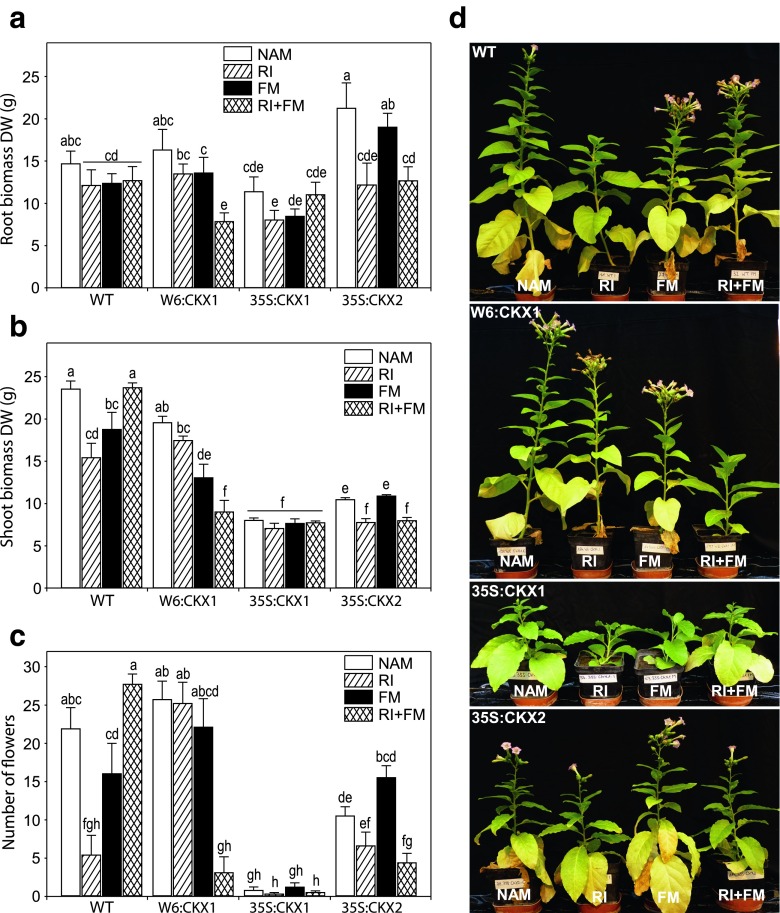
Influence of arbuscular mycorrhizal (AM) fungal inoculation on tobacco plant biomass and reproduction. The effect of the AM fungus *Rhizophagus irregularis* strain RI, strain FM, and their co-inoculation (RI + FM) on root (**a**) and shoot (**b**) dry weight (DW) and number of flowers (**c**) of 8-week-old tobacco plants were compared with the respective non-AM (NAM) plants of wild-type (WT) and transgenic lines with root-specific (W6:CKX1) or constitutive (35S:CKX1 and 35S:CKX2) reduction of cytokinin levels. Values are means + SE, *n* = 10. For each plant parameter, *bars topped by the same letter* are not significantly different (*p* < 0.05) according to Duncan’s multiple range test. **d** Representative plants that have a similar shoot phenotype as the plants used for the corresponding measurements (**a**–**c**) are shown

Next, we analyzed the influence of the AM fungal inoculations on the biomass formation of roots or shoots of transgenic and WT plants. We found that root biomass was influenced by plant genotype, by the fungal strain RI, and by the interaction of plant genotype and strain RI. Shoot biomasses, however, were influenced by all factors and their interactions (Fig. 2a, b; Supplemental Table 3). Mycorrhization with the *R. irregularis* strain FM did not affect significantly the root biomass independent of the plant genotype (Fig. 2a). Conversely, the combined inoculum of *R. irregularis* strains reduced significantly the root biomass of the plant lines with lowered CK content when these had not a strongly reduced CK level in the shoots, i.e., in W6:CKX1 and 35S:CKX2. The strain RI alone reduced also the root biomass but only of 35S:CKX2 plants. Single *R. irregularis* strains reduced the shoot biomass of WT plants but their co-inoculation had no effect (Fig. 2b, d). A reduction of the shoot biomass by the mycorrhizal fungus was also noted in transgenic plants with bioactive CK levels in shoots similar to that of WT or with only a moderate phenotype of reduced CK status in shoots, i.e., in W6:CKX1 and 35S:CKX2 (Fig. 2b, d). In contrast, the stronger reduction of shoot biomass as a consequence of the lowered levels of CK (35S:CKX1) was not reduced further by any AM inoculation compared with the NAM 35S:CKX1 plants (Fig. 2b, d). Although the co-inoculation with RI and FM neutralized their negative effects on the shoot biomass of WT plants, it reduced synergistically the shoot biomass of W6:CKX1, and maintained the RI negative effects on the shoot biomass of 35S:CKX2 (Fig. 2a, d). Overall, the different growth response to *R. irregularis* in 35S:CKX1 compared to W6:CKX1 and WT suggests that the effects of the root CK status on AM symbiotic function was dependent on the shoot CK status. Moreover, the lowered levels of CK in roots appear to increase the plants’ susceptibility to growth depression following AM fungal colonization. However, this growth depression did not become evident when the shoot itself was already strongly reduced due to lowered levels of CK.

As a proxy for plant fitness, we counted the number of flowers in 8-week-old plants. As previously reported, a lowered CK content of the shoots retards the reproductive development of tobacco plants and reduces the overall number of flowers (Werner et al. 2001), which is confirmed by the 35S:CKX1 and 35S:CKX2 NAM plants in our Fig. 2c. As for plant biomass, the effects of AM fungal inoculations on the number of flowers depended on the combination of *R. irregularis* inoculation and plant genotype (Fig. 2c; Supplemental Table 3). *R. irregularis* strain RI reduced the number of flowers of WT by 75 %, while the effects of strain FM and co-inoculation of the strains were not significant compared with the NAM WT plants (Fig. 2c). Although the single-strain inoculations had no significant effects on the number of flowers of W6:CKX1 and 35S:CKX2 (Fig. 2c), the co-inoculation with both strains reduced the number of flowers by 87 and 58 % in W6:CKX1 and 35S:CKX2 compared to their NAM control plants, respectively (Fig. 2c). In contrast, the reproductive development of 35S:CKX1 was not affected by AM fungal colonization (Fig. 2c). Taken together, the increased susceptibility of the plants to fungal parasitism observed on the plant biomass was also reflected in altered plant fitness.

### Content of phosphorus, nitrogen, and carbon in shoots

**Fig. 3:**
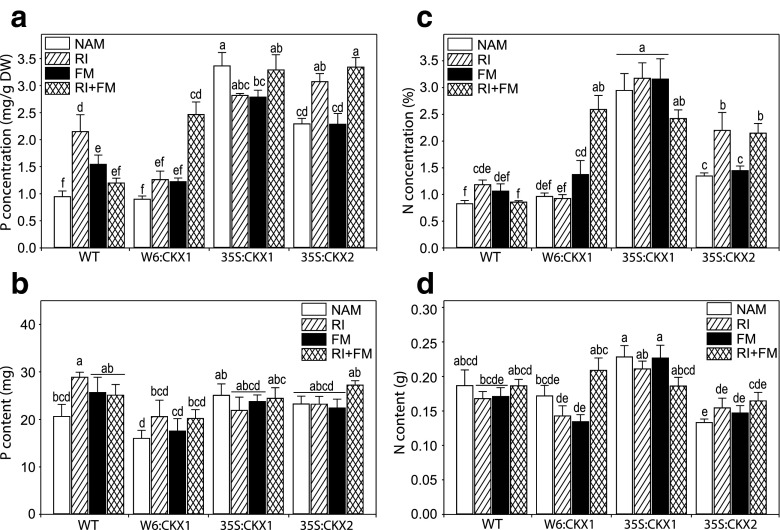
Influence of arbuscular mycorrhizal (AM) fungal inoculation on phosphorus and nitrogen in tobacco shoots. The concentration (**a**) and content (**b**) of phosphorus (P) and nitrogen (N) (**c**, **d**) was measured in shoots of 8-week-old tobacco wild-type (WT) plants and transgenic lines with root-specific (W6:CKX1) or constitutive (35S:CKX1 and 35S:CKX2) reduction of cytokinin levels and inoculated with the AM fungus *Rhizophagus irregularis* strain RI, strain FM, or their co-inoculation (RI + FM) and compared to the respective non-AM (NAM) control plants. Values are means + SE. *n* = 10 for N and *n* = 5 for P. For each parameter, *bars topped by the same letter* are not significantly different (*p* < 0.05) according to Duncan’s multiple range test

The relative availability of P, N, and C has an influence on plant growth responses to AM fungi. Therefore, we determined the P, N, and C content in shoots of AM plants and compared it with the content of the respective NAM plants. *R. irregularis* strain RI increased the concentration of P in WT shoots significantly more than strain FM, but the co-inoculation with RI and FM had no significant effect (Fig. 3c; Supplemental Table 4). Thus, only RI enhanced the total P content in shoots of WT plants (Fig. 3b; Supplemental Table 4). Although the concentration of P in shoots was increased by co-inoculation of the strains in both W6:CKX1 and 35S:CKX2 and by RI in 35S:CKX2 (Fig. 3a), these increases were associated with an unchanged total P content in shoots (Fig. 3b) due the reduction in shoot biomass (Fig. 2b). The concentration and total content of P in shoots of 35S:CKX1 remained unaltered across fungal inoculations (Fig. 3b).

The concentration of N in shoots was also altered by the AM fungal inoculations depending on the plant genotype (Fig. 3c; Supplemental Table 4). Strain RI increased the concentration of N in shoots of WT and 35S:CKX2, while the strain FM increased only the concentration of N in shoots of W6:CKX1 (Fig. 3c). Co-inoculation with RI and FM suppressed the positive effect of RI on the concentration of N in shoots of WT, enhanced synergistically the concentration of N in shoots of W6:CKX1, and maintained the positive effects of RI on the concentration of N in shoots of 35S:CKX2 (Fig. 3c). These increases, however, were associated with an unchanged total N content in shoots (Fig. 3d; Supplemental Table 4). Similarly to the results for P, the concentration and total content of N in shoots of 35S:CKX1 remained unaltered across fungal inoculations (Fig. 3c, d). Overall, the AM-mediated increases of P and N were not generally associated with increases of total uptake, except for the P benefit provided by strain RI to WT plants (Fig. 3).

**Fig. 4:**
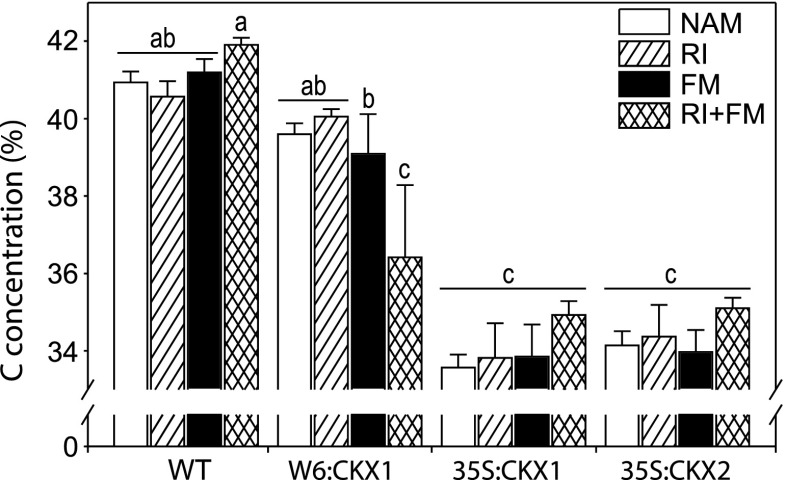
Influence of arbuscular mycorrhizal (AM) fungal inoculation on carbon concentration in shoots of tobacco plants. The concentration of carbon (C) was measured in shoots of 8-week-old tobacco wild-type (WT) plants and transgenic lines with root-specific (W6:CKX1) or constitutive (35S:CKX1 and 35S:CKX2) reduction of cytokinin levels inoculated with the AM fungus *Rhizophagus irregularis* strain RI, strain FM or their co-inoculation (RI + FM) and compared to the respective non-AM (NAM) control plants. Values are means + SE. *n* = 10. For each parameter, *bars topped by the same letter* are not significantly different (*p* < 0.05) according to Duncan’s multiple range test

We then questioned whether the depression of plant growth following *R. irregularis* colonization was associated with a reduced concentration of C in shoots. Although the NAM W6:CKX1 plants had a concentration of C in shoots similar to that of the WT (Fig. 4), the co-inoculated strains reduced synergistically by 8 % the concentration of C in shoots of W6:CKX1 compared with the NAM W6:CKX1 plants (Fig. 4). Although the concentration of C in shoots of WT plants was 17 % higher than that of 35S:CKX1 and 35S:CKX2 plants (Fig. 4), the fungal inoculations did not affect the concentration of C in shoots in none of these three genotypes compared with their respective NAM controls (Fig. 4). Therefore, the C concentrations in shoots were, in general, not affected by *R. irregularis* inoculation. An exception was the reduction following co-inoculation observed in the transgenic plants with lowered CK levels in their roots (W6:CKX1), and this reduction was associated with a strong growth depression as shown above.

### Root transcript levels of phosphate transporter genes

**Fig. 5:**
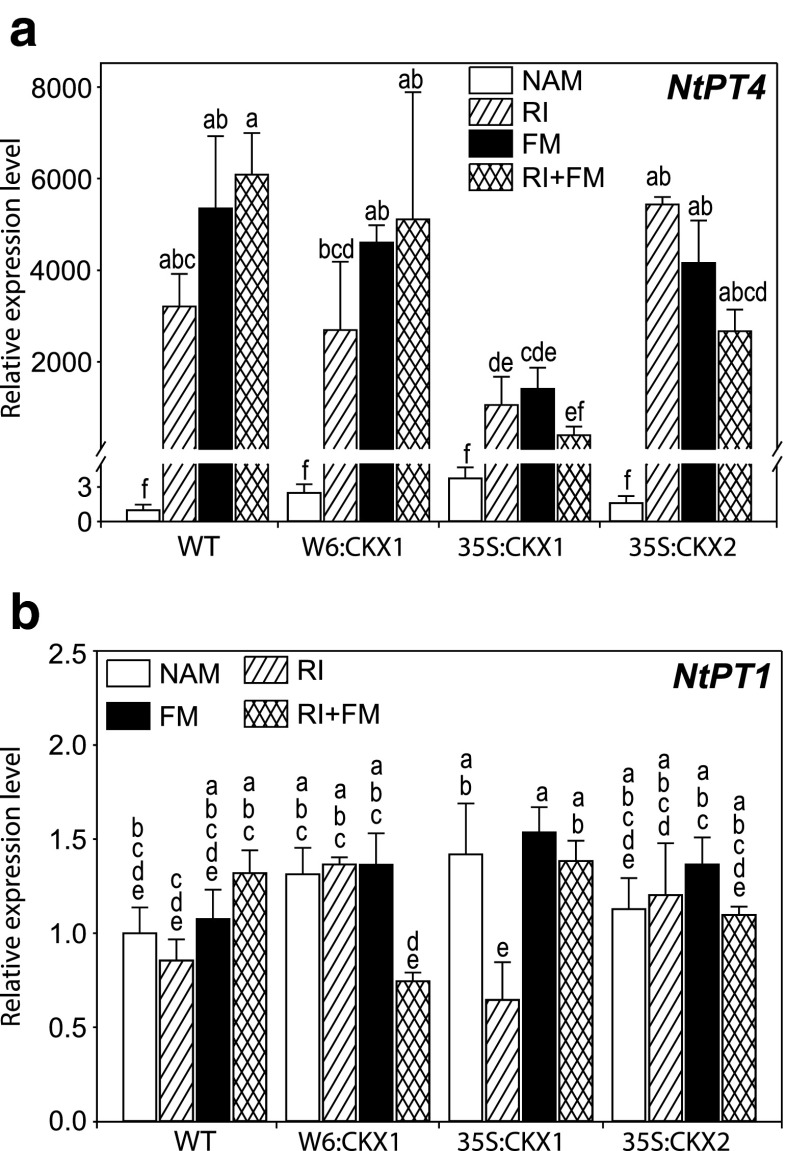
Influence of arbuscular mycorrhizal (AM) fungal inoculation on the expression of phosphate transporter genes in tobacco roots. Steady state mRNA levels of the phosphate transporter genes *NtPT4* (**a**) and *NtPT1* (**b**) were measured in roots of 8-week-old tobacco wild-type (WT) plants and transgenic lines with root-specific (W6:CKX1) or constitutive (35S:CKX1 and 35S:CKX2) reduction of cytokinin levels inoculated with the AM fungus *Rhizophagus irregularis* strain RI, strain FM, or their co-inoculation (RI + FM) and compared to the respective non-AM (NAM) control plants. Values are means + SE. *n* = 3. Each biological replicate contained roots from at least three individual plants. In both cases, the expression level of WT in non-AM controls was set to 1. For each parameter, *bars topped by the same letter* are not significantly different (*p* < 0.05) according to Duncan’s multiple range test

To test whether the altered levels of CK in plants affect the impact of *R. irregularis* on the transcript levels of *PT* genes in the roots, we determined the relative transcription levels of *NtPT4* and *NtPT1* which are involved in the mycorrhizal or the direct pathway of Pi uptake in tobacco, respectively (Chen et al. 2007). Our results confirm that *R. irregularis* induces a higher transcript level of *NtPT4*, which was increased up to 6000-fold upon fungal inoculation (Fig. 5a; Supplemental Table 5). This induction did not differ significantly among *R. irregularis* strains or their co-inoculation across the different plant lines (Fig. 5a). However, in the case of 35S:CKX1 roots, the induction of *NtPT4* transcripts following *R. irregularis* colonization was approximately 80 % lower than that of WT, W6:CKX1, and 35S:CKX2 roots (Fig. 5a). The relative transcript levels of *NtPT1* neither differed among the plant lines nor were they strongly affected by *R. irregularis* colonization, as the transcript levels varied only between 0.5- and 1.5-fold (Fig. 5b). Overall, the root CK status did not alter significantly *NtPT4* transcription, but a strong phenotype of reduced CK level in the shoots (35S:CKX1) caused a dramatic reduction of *NtPT4* induction. Furthermore, we found no strong evidence in our experiment for a suppressed direct pathway of Pi uptake following AM fungal colonization.

## Discussion

**Fig. 6:**
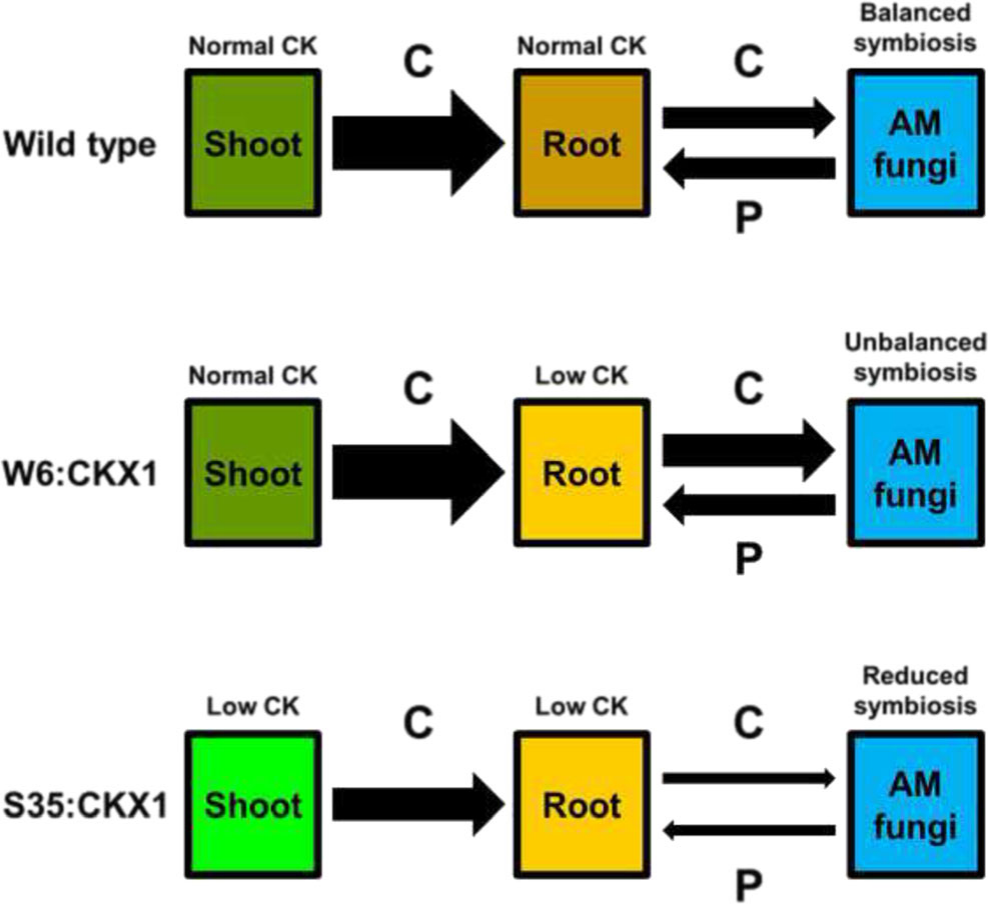
Proposed model for the cytokinin (CK) regulation of bidirectional exchange of carbon and phosphorus in arbuscular mycorrhizal (AM) symbiosis. A normal CK status in shoots and in roots contributes to balance the bidirectional flow of carbon (C) and phosphorus (P) between symbionts (balanced symbiosis), which is critical for the growth promotion of AM plants (Johnson 2010). A normal CK status of the shoots combined with reduced CK status in the roots maintain a strong source of C from the shoots into the roots (Werner et al. 2008) but may reduce the sink capacity of the roots in relation to that of the AM fungi, irrespective of P supply, causing an unbalanced C for P exchange between symbionts (unbalanced symbiosis). This can lead to fungal parasitism and consequently to reduced growth of AM plants (Johnson 2010). A strongly reduced CK status of the shoots negatively regulates the source of C from the shoots by reducing the availability of sugars (Werner et al. 2008), which may reduce the AM pathway for P uptake, irrespective of the root CK status (reduced symbiosis). *Arrow thickness* illustrates the relative flow strength of C or P

AM symbiosis is functionally important for the nutrition, growth and fitness of most terrestrial plants. Plants employ a broad phytohormone network to regulate their symbiotic interaction with AM fungi (Bucher et al. 2014; Gutjahr 2014; Pozo et al. 2015), but the regulatory role of CK is not well understood. Here, we tested the effects of root-specific and constitutive reduction of CK levels using ectopic expression of *CKX* genes in tobacco. Our data provide evidence for organ-specific effects of CK on AM symbiosis and its consequences for the plant host, and suggests that these effects are mediated through modulation of C availability to the fungus, in part independently of P and N supply (Fig. 6).

AM fungi can affect the endogenous CK levels in shoots and roots of their host plants. These effects may be either positive (Allen et al. 1980; Baas and Kuiper 1989; Danneberg et al. 1993; Drüge and Schonbeck 1992; Shaul-Keinan et al. 2002; Torelli et al. 2000; Yao et al. 2005), neutral (Danneberg et al. 1993; Jones et al. 2015), or negative (Drüge and Schonbeck 1992; Jones et al. 2015; Torelli et al. 2000). In tobacco, P supply alone had similar positive effects on the CK metabolite levels in leaves as *R. irregularis*, while in roots the fungus induced specifically a zeatin riboside and increased by 16-fold the induction of a isopentenyl adenosine compared with P supply (Shaul-Keinan et al. 2002). This suggests a high degree of AM specificity in inducing the CK levels in roots. As a lowered CK status in tobacco can increase AM hyphal colonization (Cosme and Wurst 2013), it is plausible that increased levels of CK in roots may cause a negative feedback on further fungal colonization. In our study, the constitutive reduction of CK levels in 35S:CKX1 plants caused a stronger reduction on the shoot than 35S:CKX2 and reduced the hyphal and arbuscule colonization as well as the *NtPT4* induction. However, these reductions were not observed in the W6:CKX1 plants that have a lowered CK levels restricted to their roots. This suggests that the reduced colonization and *NtPT4* induction in 35S:CKX1 was caused by the lowered CK levels in the shoots but not in the roots. Consistently, it was recently reported that the positive effects of plant CK overproduction on arbuscule colonization in pea was controlled by the shoots (Jones et al. 2015). In our study, although we observed strong plant-fungus interactions, possibly caused by specificities of plant and fungal genotypes, the use of different transgenic tobacco lines, *R. irregularis* strains, and their co-inoculation unveiled consistent functions of root CK. Root CK prevented root growth depression following *R. irregularis* colonization, restricted synergistic depressions of the plant growth, the fitness, and the shoot concentration of C following strain co-inoculation, and secured a P benefit when the symbiotic cost on shoot growth and plant fitness was strong. Taken together, our data suggests that shoot- and root-specific alterations of CK levels can both play important functions in AM symbioses.

The comparison of *R. irregularis* colonization in W6:CKX1, 35S:CKX1, and WT plants revealed a positive impact of shoot CK on AM symbiosis. This positive impact became evident through the reduced AM colonization success in 35S:CKX1 plants which differed from the two other genotypes by a stronger shoot reduction as a consequence of the lowered CK status. Consistently, Jones et al. (2015) used grafting experiments to show that the positive effects of the CK-overproducing E151 pea mutant on the number of arbuscules in roots was regulated only by the shoot CK status. In our study, the difference in AM colonization may be caused by a lower sugar availability derived from source leaves, as the leaves of 35S:CKX1 transgenic tobacco have a 30 % reduction in sugar content compared with the leaves of WT (Werner et al. 2008). In addition, 35S:CKX1 plants accumulate 80 % more starch in sink leaves than the WT (Werner et al. 2008), which represent an aboveground sink that may contribute to restrict further the fungal access to C in roots, and consequently reduce the AM symbiotic development, i.e., decrease hyphal and arbuscules colonization and *NtPT4* induction. Consistently, this C restriction protected 35S:CKX1 roots against fungal parasitism as reflected by the neutral effects on plant growth and fitness. The root transcription of *PT4* is specifically induced in root cortex cells during arbuscule formation to equip the cell membranes for Pi transport (Chen et al. 2007; Franken et al. 2007) and may require the transcription of an AM fungal *monosaccharide transporter* (*MST*) gene, which in turn is induced by sugar availability (Helber et al. 2011). Thus, an indirect negative effect of a low-shoot CK status on root *NtPT4* transcription seems likely, i.e., a reduced source of sugars in 35S:CKX1 plants could limit the induction of *NtPT4* transcription in response to AM colonization, thus reducing the AM pathway for P uptake (Fig. 6). Altogether, this suggests that the general positive effects of AM fungi on shoot CK (e.g., Allen et al. 1980) may positively feedback on the functioning of AM symbiosis, possibly through an enhanced source of C.

The lowered CK levels in roots of W6:CKX1 and 35S:CKX2 supported higher AM fungal colonization than 35S:CKX1 and were susceptible to AM-mediated root growth depression, while the WT was not. Yet, the induction of *NtPT4* transcripts was not significantly affected by root CK, as the induced levels were not significantly different among WT, W6:CKX1, and 35S:CKX2. Furthermore, the plant uptake of P and N was not limited by the AM-mediated root growth depression. A remarkable and surprising result was the genotype-specific effect of fungal co-inoculation on plant growth. Co-inoculation with RI and FM neutralized their negative effects on shoot biomass formation of WT. However, it led to a synergistic reduction of the shoot and root biomasses and C concentration in shoots of *W6*:*CKX1*, which suggests that a lowered CK status confined to the roots may facilitate fungal acquisition of C independently of fungal P supply (Fig. 6). In *M. truncatula*, an extensive growth of intercellular hyphae in roots of *della1*/*della2* mutants and GA-treated WT without formation of arbuscules also suggested that fungal acquisition of C was independent of fungal P supply (Floss et al. 2013). Kiers et al. (2011) suggested that plants can detect, discriminate, and reward the best AM fungal partner with more C to stabilize cooperation in AM symbiosis. Our study, as well as the one of Floss et al. (2013), indicates that individual phytohormones regulate differently distinct components of this nutrient exchange. As the levels of these phytohormones are altered in response to the ever-changing environment (Pozo et al. 2015), this could explain in part why the controlled reciprocal rewards model proposed by Kiers et al. (2011) cannot always be applied (Walder and van der Heijden 2015). Consistent with our results, the levels of CK in roots of the hyper-mycorrhizal CK-overproducing E151 pea mutant dropped down to levels similar to the WT after inoculation (Jones et al. 2015). This might explain also why the lowered CK levels in 35S:CKX2 plants led to enhanced AM hyphal colonization in roots (Cosme and Wurst 2013). Indirect evidence has suggested that the intraradical hyphae can take up C in roots independently from the formation of arbuscules (Smith and Read 2008). Moreover, in contrast to the intracellular arbuscules, the membranes of the intercellular hyphae have a high ATPase activity and are therefore energized for active transport of sugars (Gianinazzi-Pearson et al. 1991) and can express a *MST* gene (Helber et al. 2011). On the other hand, although the lowered CK status in tobacco roots increased root growth rates (Werner et al. 2001, 2010), their sugar content was reduced presumably due to the rapid metabolic utilization for growth (Werner et al. 2008). This implies that transfer of sugars from the plant to the AM fungus in roots with a lowered CK status is most likely mediated by active hyphal transport, with an AM fungal sink competing effectively with the sink systems of the host plant. Thus, an AM-specific increase of CK levels in tobacco roots (Shaul-Keinan et al. 2002) might be involved in enhancing the C sink capacity of the plant in relation to that of the fungus, which in turn may limit hyphal proliferation in roots (Cosme and Wurst 2013) or avert fungal parasitism.

The relation between CK and growth of AM plants has been so far unclear. A strong correlation between increased CK levels and improved photosynthesis and growth of AM plants led to the hypothesis that CK is part of the positive AM effect on plant performance (Allen et al. 1980; Drüge and Schonbeck 1992). However, this hypothesis has been disputed as a correlation was not always observed (Baas and Kuiper 1989; Danneberg et al. 1993). The AM fungi, however, do not always increase simultaneously the CK levels in shoots and roots and may even reduce it in roots under specific conditions. The CK levels might decrease in AM roots under high P amendment (Torelli et al. 2000) or temporarily during early colonization (Drüge and Schonbeck 1992), which is often associated with plant growth depression (Johnson 2010; Smith and Read 2008). Neutral growth responses in AM plants have been associated with elevated root CK levels combined with small or no changes in shoot CK content (Baas and Kuiper 1989; Danneberg et al. 1993; Shaul-Keinan et al. 2002). A stronger increase of the shoot CK content accompanied by elevated root CK levels were associated with a positive plant growth response to AM symbiosis (Allen et al. 1980; Drüge and Schonbeck 1992; Yao et al. 2005). Our study suggests that both shoot- and root-specific alterations of CK levels play important roles in the relation between CK homeostasis and growth of AM plants. The shoot CK seems to affect positively AM functioning, potentially enhancing the AM pathway for P uptake, while root CK avert fungal parasitism, possibly by limiting the relative C sink capacity of the fungus. Therefore, the general positive effects of AM fungi on the CK levels in both shoots and roots (e.g., Allen et al. 1980) might be required to balance the C for P exchange between symbionts (Fig. 6) and consequently for the growth promotion of AM plants. By using root-specific and constitutive expression of *CKX* genes, our study provides a model (Fig. 6) illustrating how plants may employ CK in shoots and roots as part of their regulatory network to fine-tune the bidirectional exchange of nutrients with ubiquitous AM fungi.

## Electronic supplementary material

Below is the link to the electronic supplementary material.

Supplemental Table 1 (PDF 385 kb)

Supplemental Table 2 (PDF 155 kb)

Supplemental Table 3 (PDF 89 kb)

Supplemental Table 4 (PDF 81 kb)

Supplemental Table 5 (PDF 89 kb)
