# Supplementary material for: Shoot- and root-borne cytokinin influences arbuscular mycorrhizal symbiosis
Source: Mycorrhiza. 2016 May 19;26(7):709–20. doi: 10.1007/s00572-016-0706-3 (PMC5034000; doi:10.1007/s00572-016-0706-3)
Supplement: Supplementary file 1 — (PDF 385 kb) [file 572_2016_706_MOESM1_ESM.pdf]

Supplemental Table 1. Primers used for quantitative real-time RT-PCR.

**Gene name**	**Gene bank accession No.**	**Primer sequence**
NtEF-1α	AF120093	5'- TGAGATGCACCACGAAGCTC -3'<br>5'- CCAACATTGTCACCAGGAAGTG -3'
NtL25	L18908	5'- CCCCTCACCACAGAGTCTGC -3'<br>5'- AAGGGTGTTGTTGTCCTCAATCTT -3'
NtPT1	AB020061	5'- AGCGTTCATTGCTGCTGTTT -3'<br>5'- AGAGCGTCGGCATGATATGT -3'
NtPT4	EF091672	5'- GTCAACTCGTGGGGCGTTTAT -3'<br>5'- CTCAGGCTCCGTGGACAAAAT -3'
