# Supplementary material for: Shoot- and root-borne cytokinin influences arbuscular mycorrhizal symbiosis
Source: Mycorrhiza. 2016 May 19;26(7):709–20. doi: 10.1007/s00572-016-0706-3 (PMC5034000; doi:10.1007/s00572-016-0706-3)
Supplement: Supplementary file 2 — (PDF 155 kb) [file 572_2016_706_MOESM2_ESM.pdf]

Supplemental Table 2. Results of two-way ANOVAs on AM hyphae and arbuscules colonization in tobacco roots with the categorical factors tobacco lines (four levels: WT, W6:CKX1, 35S:CKX1 or 35S:CKX2) and AM inoculation (three levels: strain RI, FM or both RI and FM). For the mean values see Fig. 1.

Factors		Hyphae		Arbuscules	
	df	*F*	*P*	*F*	*P*
Tobacco lines (T)	3	**32.7**	*******	**9.2**	*******
AM inoculation (AM)	2	*2.6*	(*)	0.8	
T x AM	6	1.1		*2.0*	(*)
Residuals	108				

AM, arbuscular mycorrhizal. WT, wild type. df, degrees of freedom.

For $P < 0.05$, 0.01 and 0.001, significance levels of F values are presented as *, ** and ***, respectively, and are in bold. F values accompanied by (*) are marginally non-significant ($P < 0.100$) and are in italic.
