# Supplementary material for: Shoot- and root-borne cytokinin influences arbuscular mycorrhizal symbiosis
Source: Mycorrhiza. 2016 May 19;26(7):709–20. doi: 10.1007/s00572-016-0706-3 (PMC5034000; doi:10.1007/s00572-016-0706-3)
Supplement: Supplementary file 3 — (PDF 89 kb) [file 572_2016_706_MOESM3_ESM.pdf]

Supplemental Table 3. Results of three-way ANOVAs on root and shoot DW and on number of flowers in tobacco plants with the categorical factors tobacco lines (four levels: WT, W6:CKX1, 35S:CKX1 or 35S:CKX2), *R. irregularis* RI (two levels: -, +), and *R. irregularis* FM (two levels: -, +). For the mean values see Fig. 2.

Factors		Root DW		Shoot DW		Number of flowers	
	df	*F*	*P*	*F*	*P*	*F*	*P*
Tobacco line (T)	3	**10.8**	***	**132.2**	***	**59.8**	***
R. irregularis RI	1	**18.1**	***	**23.0**	***	**29.2**	***
R. irregularis FM	1	2.6		**8.7**	**	1.0	
T x RI	3	**3.3**	*	**2.7**	*	**3.6**	*
T x FM	3	*2.6*	(*)	**20.3**	***	**15.7**	***
RI x FM	1	1.7		**5.2**	*	0.1	
T x RI x FM	3	*2.5*	(*)	**8.6**	***	**19.4**	***
Residuals	144						

DW, dry weight. WT, wild type. df, degrees of freedom.

For *P* < 0.05, 0.01 and 0.001, significance levels of *F* values are presented as *, ** and ***, respectively, and are in bold. *F* values accompanied by (*) are marginally non-significant and are in italic.
