# Supplementary material for: Shoot- and root-borne cytokinin influences arbuscular mycorrhizal symbiosis
Source: Mycorrhiza. 2016 May 19;26(7):709–20. doi: 10.1007/s00572-016-0706-3 (PMC5034000; doi:10.1007/s00572-016-0706-3)
Supplement: Supplementary file 4 — (PDF 81 kb) [file 572_2016_706_MOESM4_ESM.pdf]

Supplemental Table 4. Results of three-way ANOVAs on concentration of P, N and C and content of P and N in shoots with the categorical factors tobacco lines (four levels: WT, W6:CKX1, 35S:CKX1 or 35S:CKX2), *R. irregularis* RI (two levels: -, +), and *R. irregularis* FM (two levels: -, +). For the mean values see Fig. 3.

Factors	P concentration			P total content			N concentration		N total content		C concentration	
	df	*F*	*P*	df	*F*	*P*	*F*	*P*	*F*	*P*	*F*	*P*
Tobacco line (T)	3	**104.5**	***	3	**7.0**	***	**111.5**	***	**12.2**	***	**69.7**	***
R. irregularis RI	1	**42.2**	***	1	*4.0*	(*)	**19.0**	***	0.0		0.0	
R. irregularis FM	1	**5.5**	*	1	0.6		**9.0**	**	0.2		0.1	
T x RI	3	**6.0**	**	3	1.2		**6.2**	***	*2.6*	(*)	1.4	
T x FM	3	**7.4**	***	3	0.1		**14.9**	***	0.7		**3.3**	*
RI x FM	1	0.0		1	0.1		0.1		*3.8*	(*)	0.0	
T x RI x FM	3	**13.8**	***	3	2.0		**10.0**	***	**3.8**	*	1.9	
Residuals	64			144								

P, phosphorus. N, nitrogen. C, carbon. WT, wild type. df, degrees of freedom.

For $P < 0.05$, 0.01 and 0.001, significance levels of *F* values are presented as *, ** and ***, respectively, and are in bold. *F* values accompanied by (*) are marginally non-significant and are in italic. $n = 5$ for P and $n = 10$ for N and C.
