# Supplementary material for: Shoot- and root-borne cytokinin influences arbuscular mycorrhizal symbiosis
Source: Mycorrhiza. 2016 May 19;26(7):709–20. doi: 10.1007/s00572-016-0706-3 (PMC5034000; doi:10.1007/s00572-016-0706-3)
Supplement: Supplementary file 5 — (PDF 89 kb) [file 572_2016_706_MOESM5_ESM.pdf]

Supplemental Table 5. Results of three-way ANOVAs on *NtPT4* and *NtPT1* transcript levels with the categorical factors tobacco lines (four levels: WT, W6:CKX1, 35S:CKX1 or 35S:CKX2), *R. irregularis* RI (two levels: -, +), and *R. irregularis* FM (two levels: -, +). For the mean values see Fig. 5.

Factors		*NtPT4*		*NtPT1*	
	df	*F*	*P*	*F*	*P*
Tobacco line (T)	3	**11.9**	***	1.2	
R. irregularis RI	1	**22.4**	***	**7.9**	**
R. irregularis FM	1	**55.3**	***	2.6	
T x RI	3	1.9		2.2	
T x FM	3	**4.0**	*	**4.4**	*
RI x FM	1	**43.6**	***	0.0	
T x RI x FM	3	1.2		**4.2**	*
Residuals	30				

NtPT4, *Nicotiana tabacum phosphate transpoter4*. *NtPT1*, *Nicotiana tabacum phosphate transpoter1*. WT, wild type. df, degrees of freedom.

For $P < 0.05$, 0.01 and 0.001, significance levels of *F* values are presented as *, ** and ***, respectively, and are in bold. *F* values accompanied by (*) are marginally non-significant and are in italic.
